# Deformation-Induced Crystallization Behavior of Isotactic Polypropylene Sheets Containing a β-Nucleating Agent under Solid-State Stretching

**DOI:** 10.3390/polym12061258

**Published:** 2020-05-30

**Authors:** Huajian Ji, Xulin Zhou, Xin Chen, Haili Zhao, Yu Wang, Huihao Zhu, Yulu Ma, Linsheng Xie

**Affiliations:** School of Mechanical and Power Engineering, East China University of Science and Technology, Shanghai 200237, China; shxiaoji@163.com (H.J.); 15527377408@163.com (X.Z.); chenxin198905@aliyun.com (X.C.); zhl419wsm@163.com (H.Z.); apirl.1992@outlook.com (Y.W.); zhhxhhy1994@126.com (H.Z.); myl@ecust.edu.cn (Y.M.)

**Keywords:** solid-state stretching, β-modification iPP sheet, phase transformation, cylindrites

## Abstract

The deformation-induced crystallization of an isotactic polypropylene (iPP) sheet containing a β-nucleating agent was evaluated. The phase transformation of the β-modifications was investigated and the crystal morphology was observed at room temperature after stretching at different temperatures. The results showed that the crystallinity increased after solid-state stretching. When the stretching temperature was below the initial crystallization temperature, stretching deformation promoted the orientation of amorphous molecular chains. When the deformation temperature exceeded the crystallization temperature, part of the β-modifications underwent a phase transformation process and was stretched into a shish-kebab structure. However, once the stretching temperature was close to the melting point, the β-modifications melted and recrystallized, and the shish-kebab structure underwent stress relaxation due to poor thermal stability, transforming into α-modifications. It was revealed that the crystal phase transformation mechanism of the β-modifications was based on the orientation of the molecular chains between the adjacent lamellae. In addition, the shish-kebab cylindrite structure played an important role in modifying the tensile and impact properties of the iPP sheet. The tensile and impact strengths increased by as much as 34% and 126%, respectively.

## 1. Introduction

As a general plastic, isotactic polypropylene (iPP) is mainly used in films, packaging, and the automotive industry [1,2,3,4]. iPP has four crystalline structures: α-modifications, β-modifications, γ-modifications, and smectic modifications [5,6,7]. α-modification iPP has excellent tensile strength but poor toughness [8], while β-modification iPP has better thermal properties and toughness [9]. Thus far, many researchers have studied the crystallization behavior of iPP containing a β-nucleating agent, and reported that the growth rate of the β-modification was 70% higher than that of the α-modification [10,11,12,13]. Varga and Menyhárd [14,15] proved that the nucleating agent could affect the molecular structure of iPP, and revealed that multiple β-modification morphologies existed in iPP crystalline modifications. γ-modification iPP is induced by the application of high pressure during the crystallization process [16]. Lezak et al. [17] studied the morphology and deformation behavior of iPP homopolymer containing γ-modifications obtained from isothermal crystallization at a high pressure of 200 MPa. Mostly, γ-modification iPP exists under high pressure—the higher the pressure, the larger the fraction of γ-modifications [18].

Polymer processing endows polymer products with multiple microstructures, owing to the chain orientation and multiphases. These microstructures regulate the macroscopic mechanical performance, which eventually affects the service life of the products. β-modification iPP exhibits capability of transforming the β-phase into another crystalline phase [19,20]. Li et al. [21] observed that the β–α transformation in the neck region and the crystallinity in their samples were nearly constant for engineering strains below 100%, which then slowly decreased for larger strains. Ruiying Bao et al. [22] studied the deformation-induced structure evolution of iPP. They suggested that β–α transformation was induced by intra-lamellar slips. As the deformation temperature increased, the β-modifications were reoriented by stretching. Riekel and Karger-Kocsis [23] also revealed that β-modifications were transformed into oriented α-modifications under deformation-induced processing. Shi et al. [19] also claimed that β-modifications were transformed into a smectic crystal when the stretching temperature was below 80 °C; however, they transformed into stable α-modifications at higher temperatures. Lezak et al. [24] studied the evolution of the lamellar structure of β-modification iPP. The authors suggested that inter-lamellar slips promoted the fragmentation of lamellae under higher strain, and rotation of the unconstrained fragmentation crystallites occurred along the stretching direction. However, there is still some uncertainty in the true process of β-phase transformation due to insufficient experimental evidence [25].

The stretching deformation range of the sheet is much lower than that of the film. At present, there are more studies on iPP films subjected to high deformations, but fewer studies on iPP sheets subjected to low deformations. Updated synchrotron radiation X-ray diffraction is equipped to study the deformation-induced crystallization behavior in β-modification iPP [26,27]. Sheng et al. [3] researched the mechanical properties of iPP sheets by adding a nucleating agent, and suggested that the impact strength was enhanced, but that the tensile strength was not significantly improved.

In this work, the β-nucleating agent TMB-5 was used to prepare a β-modification iPP sheet. The evolution of the microstructure of the iPP sheet at different stretching temperatures was investigated. In our previous work [28], we studied the evolution process of the α-modification lamellae phase transition of pure iPP during solid-state stretching. This work elucidated the temperature-related β-modification lamellae structural transformation and deformation-induced mechanism of β-modification iPP. It also clarifies the regulating mechanism of the microstructure on the mechanical properties. Additionally, it is an effective method for preparing both excellent tensile and impact properties.

## 2. Materials and Methods

### 2.1. Materials and Sample Preparation

iPP (1100N; M_n_ 46,000 kg·mol^−1^ and M_w_ 317,000 kg·mol^−1^) with a melt flow rate (MFR) of 12.8 g/10 min (230 °C/2.16 kg) was supported by Shenhua Group Corporation Ltd., Yinchuan, China. T_m_ = 165 °C. The β-nucleating agent TMB-5 (N,N′-dicyclohexyl-2,6-naphthalenedicarboxamide) was provided by Shanxi Chemical Industry Research Institute, Xian, China.

iPP and TMB-5 were mixed using a twin-rotor continuous mixing extruder (Laboratory homemade). The barrel temperature was 200 °C. The content of TMB-5 was 0.02 wt%. The pelletized iPP containing the β-nucleating agent was injection-molded into an iPP sheet sample (180 mm in length, 15 mm in width, and 3.5 mm in thickness) using an injection-molding machine (COSMOS 80, Ge Lan Manufacture Co., Ltd., Wuxi, China). The injection temperature was 200 °C. Samples were stretched using the following experimental protocol: (1) The sheets were clamped by a tensile testing machine with a high-temperature test chamber; (2) the sample was heated from room temperature to testing temperature (i.e., 110 °C, 120 °C, 130 °C, 140 °C, and 150 °C) and then was equilibrated for 15 min; (3) the distance between the clamps was set to 100 mm and the heated sample was stretched to the set stretching strain value (i.e., 5%, 10%, 15%, and 20%) at a stretching rate of 3 mm/min by a stretching fixture; (4) the stretched iPP sheet with a fixed length was cooled to room temperature for testing and characterization.

### 2.2. Characterization

The X-ray diffraction (XRD) experiment was carried out on a Rigaku D/max 2550VB/PC X-ray diffractometer. The diffraction angle 2θ was measured from 3° to 40° The relative content of the β-modification iPP, K_β_, was calculated by Equation (1) [16,29]:

(1) $$K_{\beta }=\frac{A_{\beta }\left(110\right)}{A_{\beta }\left(110\right)+A_{\alpha }\left(110\right)+A_{\alpha }\left(040\right)+A_{\alpha }\left(130\right)},$$

where *A*_β_ (110) is the area of the β-modification diffraction angle at 16.1° and *A*_α_ (110), *A*_α_ (040), and *A*_α_ (130) are the areas of the α-modification diffraction angles at 14.1°, 16.9°, and 18.5°, respectively.

The melting characteristics of the samples were determined by a TA-Q200 instrument. The experiment was performed in a range from 20 to 200 °C. The total crystallinity, X_c_, for both α- and β-modifications could be obtained from Equation (2):

(2) $$X_{C}=X_{\alpha }+X_{\beta },$$

where X_α_ and X_β_ are the crystallinity of the α- and β-modifications, and can be calculated by Equation (3):

(3) $$X_{i}=\frac{\Delta H_{i}}{\Delta H_{i}^{0}}\times 100\%,$$

where $\Delta H_{i}^{}$ is the heat of fusion of the iPP sheet and $\Delta H_{i}^{0}$ is the heat of fusion of the complete crystallization of either the α- or the β-modification iPP (177 J/g and 168.5 J/g) [30].

A two-dimensional small-angle X-ray scattering (2D-SAXS) experiment was performed by a BL16B in the Shanghai synchrotron radiation facility (SSRF). A Pilatus detector (with 1475 × 1679 pixels—pixel size of 0.172 mm) was equipped to collect the scattering patterns. The sample-to-detector distance was 1841 mm. In the scattering pattern, if the pattern was isotropic or arced, the desired one-dimensional intensity distribution was obtained by multiplying I(q) by q^2^ for any radial scan. The one-dimensional intensity distribution I(q_1_) (Lorentz correction) at each q_1_ was calculated by using Equation (4) [31,32]:

(4) $$I(q_{1})=q^{2}\times I\left(q\right),$$

where the scattering vector q = 4π (sin θ) /λ, 2θ is the scattering angle, λ = 0.124 nm.

The sizes of crystal could be measured from the one-dimensional correlation function K(z), which was obtained by Equation (5) [32]:

(5) $$K(z)=\frac{\int_{0}^{\infty }I(q_{1})\cos(q_{1}z)dq_{1}}{\int_{0}^{\infty }I(q_{1})dq_{1}},$$

where z denotes the stretching direction.

To observe the crystal morphology, the fracture surfaces of the samples were etched using a solution of sulfuric acid and phosphoric acid, and adding potassium permanganate for 24 h according to the method described by Olley [33]. The micromorphology was observed by a scanning electron microscope (S3400, Hitach, Tokyo, Japan), which had an accelerating voltage of 15 kV. All surfaces were sputtered with gold before the test. 

The tensile and notched impact strength of the iPP sheet were determined by a Universal Testing Machine (RGM-2020, Shenzhen Reger Instrument, Shenzhen, China) and an Impact Testing Machine (PTM1100-B1, SUNS, Shenzhen, China), respectively. The sizes of the samples were prepared according to ASTM D638 and ASTM D256. At least five tests per data point were conducted.

## 3. Results and Discussion

### 3.1. X-ray Diffraction (XRD)

**Figure 1:**
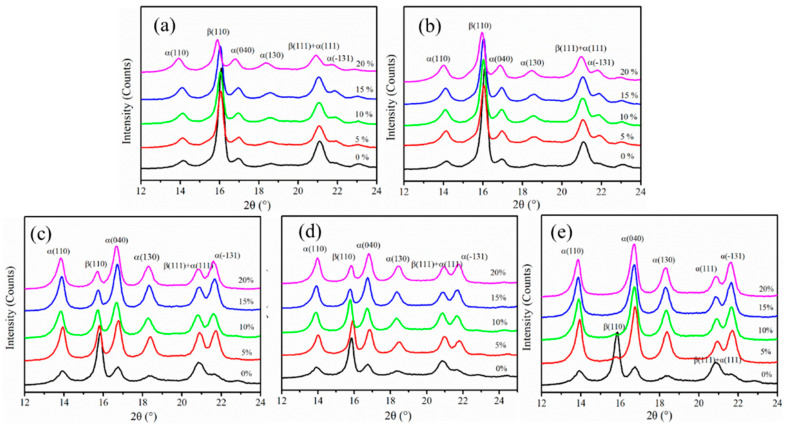
X-ray diffraction (XRD) curves of the isotactic polypropylene (iPP) sheet after stretching at different temperatures: (**a**) 110 °C, (**b**) 120 °C, (**c**) 130 °C, (**d**) 140 °C, and (**e**) 150 °C.

The crystal structure of the iPP sheet is usually determined upon analysis of the XRD diffraction peaks. β(110) and β(111) are the β-modification diffraction peaks [16]. The XRD diffraction patterns of the iPP sheet at different stretching temperatures are shown in Figure 1. In Figure 1a,b, the β-modifications dominated in the crystal region of the untreated iPP sheet, and the diffraction peak intensity of the β-modifications of the stretched iPP sheet gradually decreased with an increase in the stretching strain. When the stretching temperature was below the crystallization temperature (i.e., 124 °C), the external force did not overcome the internal friction of the molecular chain movement of the lamellae. Deformation of the β-modification lamellae was difficult. As the stretching strain increased, part of the randomly oriented β-modification lamellae was damaged during the solid-state stretching process. Therefore, the diffraction peak intensity of β(110) decreased.

In Figure 1c,d, it can be seen that the α-modifications dominated in the crystal region when the stretching temperature exceeded the crystallization temperature of the iPP sheet. The diffraction peak intensities of the α-modifications increased with an increase in the stretching strain, implying that transformation of the β–α modifications had occurred. It could also be found from Figure 1c,d that the α-modification diffraction peak intensities did not increase when the stretching strain exceeded 15%, which indicates that the β–α phase transformation process was not dominant. In addition, the intensity of the β-modification diffraction peak was substantially unchanged, which implies that the β-modifications in the iPP sheet were not completely converted into α-modifications. When the solid-state stretching temperature was higher (i.e., above the crystallization temperature), the molecular chain movement ability increased, which resulted in β-modification lamellae slipping and transforming into a row of β-nuclei. The molecular chains of the crystal phase grew on the surface of the thermodynamically metastable β-nuclei to form a stable α-modification structure. When the stretching strain exceeded a critical value, the intra-lamellae molecular chains underwent stress relaxation, and the nucleus disappeared again [22]. This phenomenon was consistent with the recrystallization of iPP.

In Figure 1e, the intensity of the dominating β-modification diffraction peak, β(110), significantly decreased with the increase in the stretching strain, while the intensity of the α-modification diffraction peaks increased relative to the unstretched β-modification iPP sheet. The β-modifications were more likely to melt to recrystallize under external force when the stretching temperature was 150 °C (i.e., near the melting point of the β-modifications). Furthermore, oriented α-nucleation occurred, which increased the content of α-modifications in the iPP sheet.

In summary, the β–α phase transformation process occurred in the β-modification iPP sheet after solid-state stretching below the melting point. When the stretching temperature was too low, the movement ability of the molecular chains was poor, and the phase transformation process did not occur. When the stretching temperature was too high, β-modification lamellae underwent thermodynamic relaxation and were converted into α-modification lamellae. Once the stretching strain exceeded a critical strain value of 15%, β–α phase transformation did not continue to increase. This may be explained by the fact that at higher temperatures, the relaxation effect of the molecular chains was stronger than the orientation effect at a higher stretching strain, and the entanglements of the iPP molecular chains prevented the phase transformation process. 

**Figure 2:**
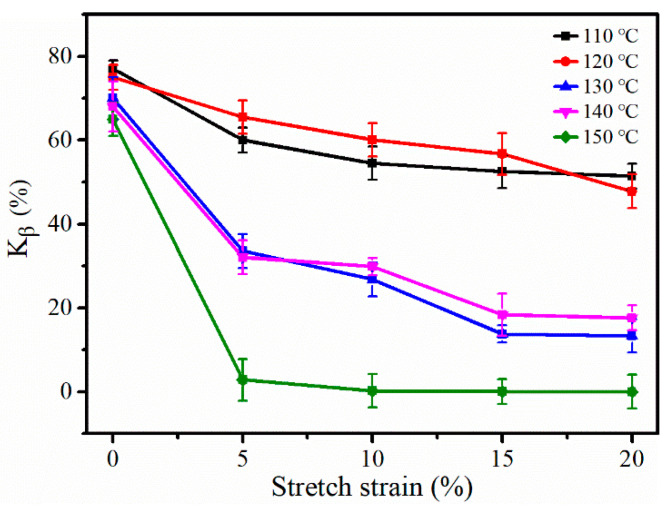
The K_β_ of the iPP sheet after stretching at different temperatures.

The relative fraction of β-modifications (K_β_, obtained from Equation (1)) of the iPP sheet are shown in Figure 2. It was known that K_β_ decreased with an increase in the stretching temperature. When the stretching temperature was lower, K_β_ decreased slowly because fewer β-modification lamellae were damaged by stretching. When the stretching temperature was above the crystallization temperature (i.e., 124 °C), K_β_ also showed a downward trend. Therefore, the phase transformation process occurred, owing to the slippage and rearrangement of the molecular chains of the lamellae. The change in K_β_ was small when the stretching strain was between 15% and 20%, which can be explained by the stress relaxation accelerating the slippage of the newly formed α-modifications. However, K_β_ rapidly decreased to near zero once the stretching temperature increased to 150 °C, during which time, a large number of α-nuclei were generated. Subsequently, the molecular chains were folded on its surface to produce α-modifications.

### 3.2. Differential Scanning Calorimetry (DSC)

**Figure 3:**
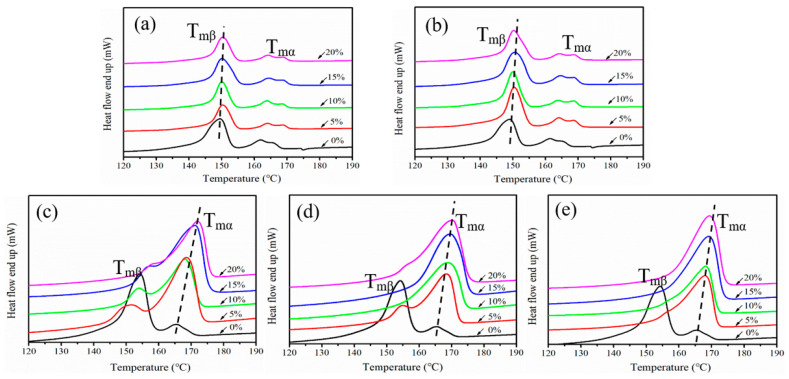
The melting curves of the iPP sheet after stretching at different temperatures: (**a**) 110 °C, (**b**) 120 °C, (**c**) 130 °C, (**d**) 140 °C, and (**e**) 150 °C.

The melting characteristics of the iPP sheet at different stretching temperatures are shown in Figure 3. The endothermic peak located at 150 °C was the melting point of the β-modifications (T_mβ_), while that located at 165 °C was the melting point of the α-modifications (T_mα_) [28]. In Figure 3a,b, the melting enthalpy of the α- and β-modifications did not change much after solid-state stretching. This can be explained by the fact that the solid-state stretching effect was not large enough to induce the phase transformation process at lower stretching temperatures. However, in Figure 3c,d, the crystal structure of the iPP sheet was mainly that of the α-modifications when the stretching temperature exceeded the recrystallization temperature. Furthermore, the melting peak of the β-modifications decreased until it eventually disappeared. The β-modification lamellae were stretched and slipped, owing to the thermodynamic metastability; thus, β–α phase transformation was accelerated. Figure 3c,d also shows that the melting points of the α- and β-modifications moved in the high-temperature direction after solid-state stretching, implying that the amorphous molecular chains were stretched.

The melting curve of the iPP sheet at a stretching temperature of 150 °C is shown in Figure 3e. It was difficult to observe the melting peak of the β-modifications, indicating that almost all the β-modifications were transformed into thermodynamically stable α-modifications. The higher stretching temperature accelerated the movement of intra-lamellae molecular chains, which was favorable for the conformation of the α-modifications.

**Figure 4:**
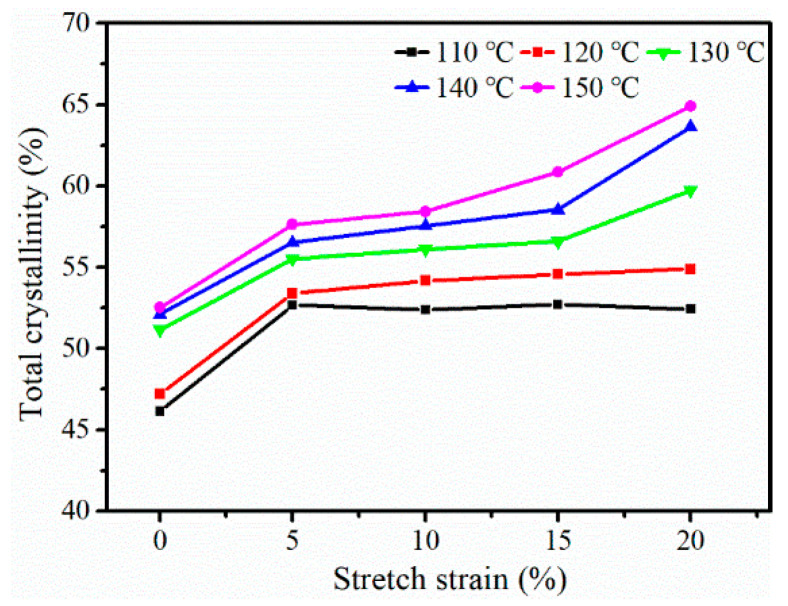
Total crystallinity of the iPP sheet after stretching at different temperatures.

For semi-crystalline polymers, crystallinity is an important factor for the properties of the material [34]. The total crystallinity (X_c_, obtained from Equations (2) and (3)) of the iPP sheet at different stretching temperatures is shown in Figure 4. The X_c_ increased with an increase in the stretching temperature because of the oriented molecular chains. While the stretching temperature was below the crystallization temperature, the phase transformation process did not occur due to the large internal friction of the movement of the lamellae. Therefore, X_c_ did not change greatly with an increase in the stretching strain. When the stretching temperature was 130~140 °C, according to the previous results, the β-modifications gradually transformed into α-modifications. As the stretching strain was increased from 5% to 15%, X_c_ increased slowly, implying that the external force work mainly promoted the crystal phase transformation and had little effect on the crystallinity. When the stretching strain was 20%, the degree of the molecular chain orientation of the iPP sheet increased, which promoted the process of crystallization. When the stretching temperature was 150 °C, the transformation from β-modifications to α-modifications resulted in an increase in the crystallinity.

### 3.3. 2D Small-Angle X-ray Scattering (2D-SAXS)

**Figure 5:**
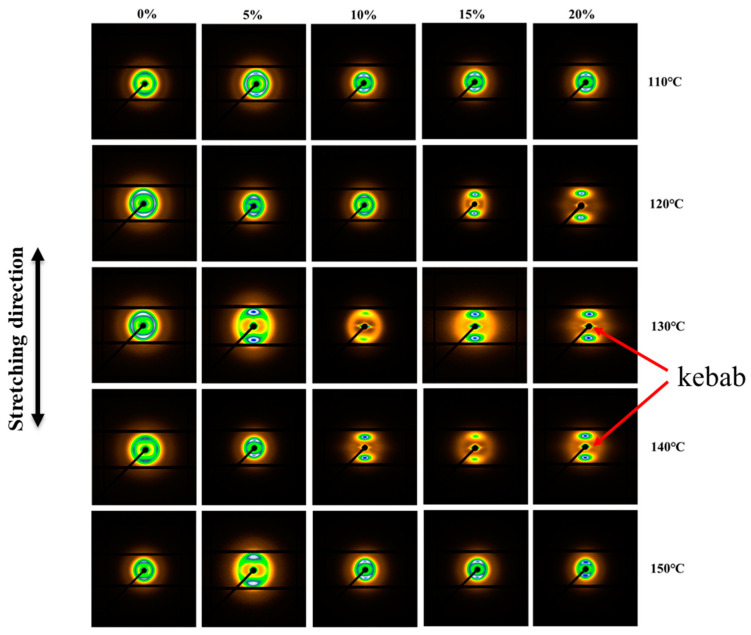
Two-dimensional small-angle X-ray scattering (2D-SAXS) patterns of the iPP sheet after stretching at different temperatures.

According to the results of XRD and DSC, solid-state stretching is able to induce the phase transformation process of an iPP sheet, but is unable to explain the orientation change of the crystal lamellae. The 2D-SAXS patterns during uniaxial stretching of the iPP sheet are shown in Figure 5. When the stretching temperature was below the crystallization temperature, the scattering patterns had no orientation under 10% stretching strain, implying that isotropic β-modifications dominated in the matrix. As the stretching strain increased, the difference in the electron densities along the stretch direction increased, which indicates that the intra-lamellae molecular chains were stretched. As a result, oriented β-modifications were generated. In addition, a scattering peak perpendicular to the stretching direction was not found, indicating that the phase transformation process did not occur. This result can be explained by the fact that the extended inter-lamellae molecular chains relaxed rapidly due to the short time of the external force action. At that moment, the external force was insufficient to change the crystal lamellae structure of the iPP sheet.

When the stretching temperature was 130~140 °C, an “inverted triangle” peak (marked by red arrows) was also observed, which suggests that a cylindrite structure of stable orientation existed in the iPP sheet. Because the orientation of the amorphous molecular chains had restricted movement and formed a “kebab” structure, the β-modification lamellae were broken and formed a “shish” structure along the stretching direction [35]. However, when the stretching temperature was 150 °C, the amorphous molecular chain had a large space for movement due to the destruction of a large number of lamellae, and the number of nucleation sites increased. As a result, shish-kebab cylindrite structures were no longer observed due to large amount of recrystallization at the elevated temperature. Hobeika et al. [36] studied the change of the lamellae structure of HDPE (High Density Polyethylene) during the temperature-deformation process. The critical strain point of the lamellae slippage was obtained by using the true stress–strain curve. The results showed that the yield strain was always around 0.12 at different temperatures or strain rates. The premise of the lamellae phase transformation process was that the lamellar could be stretched and slipped. In Figure 5, an orientation diffraction peak perpendicular to the stretching direction appeared after solid-state stretching of 10% strain at 130 °C and 140 °C. There were two reasons for this: on the one hand, the stretching temperature was higher than the iPP crystallization temperature (i.e., 124 °C), and the stability of the molecular chains of the lamellae was poor; on the other hand, the stretching strain was defined based on the displacement of the fixture movement. If it was converted into true stress–strain, the strain would exceed 10%.

The evolution of the microstructure of the β-modifications mainly depended on the stretching temperature. When the temperature was lower, the molecular chains were poorly oriented. When the temperature exceeded the crystallization temperature, the phase transformation process occurred, owing to the restricted movement of the molecular chains of the lamellae. A small amount of shish-kebab cylindrite structure was formed. When the stretching temperature was near the β-modification melting point, a large number of lamellae were destroyed and recrystallized. Almost all α-modifications were crystallized in the system. The shish-kebab structure collapsed due to stress relaxation to the α-modifications. Therefore, solid-state stretching did not continuously enhance the ability of the transformation of β-modifications, and the shish-kebab cylindrite structure was produced only at 130~140 °C.

**Figure 6:**
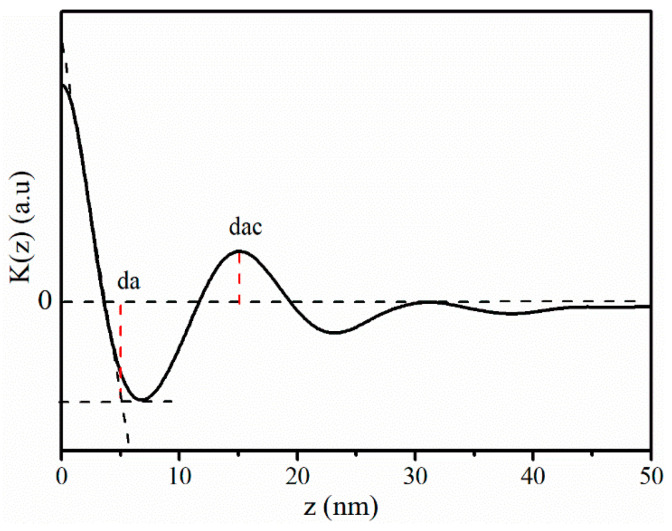
K(z) of the iPP sheet after solid-state stretching.

The above research results show that solid-state stretching can induce changes to the microstructure of the iPP sheet. To investigate the effect of solid-state stretching deformation on the sizes of the crystals, K(z) (obtained from Equations (4) and (5)) curves of the iPP sheet are shown in Figure 6. The parameters were derived from the K(z) curve (Figure 6a). The long period d_ac_ is the average spacing between lamellae, and the lamellar thickness d_c_ was obtained by subtracting the inter-lamellae coil molecular chain layer thickness from the long period. However, the crystallinity of the sample used in the present study was higher than 0.5. Thus, the smaller value d_a_ was defined as the average thickness of the inter-lamellae coil molecular chain layer [32,37].

**Figure 7:**
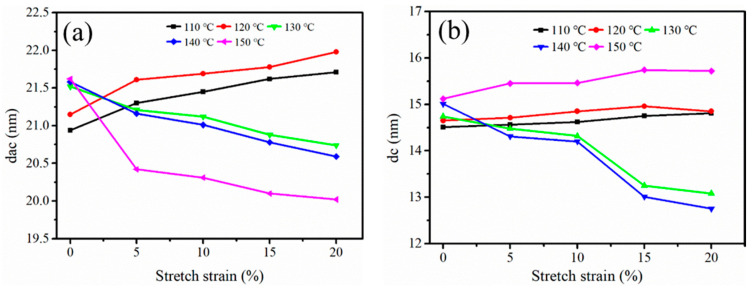
The d_ac_ and d_c_ of the iPP sheet after stretching at different temperatures.

The d_ac_ and d_c_ derived from Figure 6 are shown in Figure 7. In Figure 7a, when the stretching temperature was 110~120 °C, d_ac_ increased with increasing stretch strain. This was because the molecular chains between the adjacent lamellae were stretched. When the stretching temperature gradually increased to 130~140 °C, it was observed that the d_ac_ decreased slightly. It was clear, based on the research results in Figure 5, that a stable shish-kebab structure had been formed. Simultaneously, when the stretching strain continued to increase, the embedding of the inter-lamellae molecular chains counteracted the increased distance between adjacent lamellae, which could also explain the reduction in d_ac_. Mao et al. [38] studied the influence of the deformation induced on the d_ac_ of iPP. They exposed that the d_ac_ decreased due to densely packed lamellae, and then increased owing to the separation of lamellae. When the stretching temperature was 150 °C, d_ac_ decreased with the increasing stretch strain. This can be explained by the fact that the crystal lamellae of the iPP sheet melted and formed α-modification nucleation sites at a higher temperature, which induced the transformation of β–α modifications; α-modifications were densely packed, and the long period decreased.

The d_c_ values of the stretched iPP sheet are shown in Figure 7b. It can be found that d_c_ remained substantially unchanged when the stretching temperature was low. There was no phase transformation process because the iPP crystal lamellae were not stretched. When the stretching temperature was 130~140 °C, d_c_ decreased as the stretching strain increased from 10% to 15%. On the one hand, a shish-kebab structure was produced, which resulted in a reduction in d_c_. On the other hand, some α-modification nuclei were generated when the lamellae were broken, and the crystal nuclei crystallized into α-modifications. Therefore, it can be concluded that the application of an external force contributed substantially to the phase transformation process, rather than the simple molecular chain orientation. When the stretching temperature was 150 °C, the molecular chain was more easily stretched and oriented, which resulted in an increase in d_c_. The melting and recrystallization of the β-modifications transformed into stable α-modifications, so that the shish-kebab-like structure was absent. With an increase of the stretching strain, the external force mainly resulted in the destruction of the lamellae and produced α-nuclei. Therefore, the d_c_ increased.

### 3.4. Scanning Electron Microscopy (SEM)

**Figure 8:**
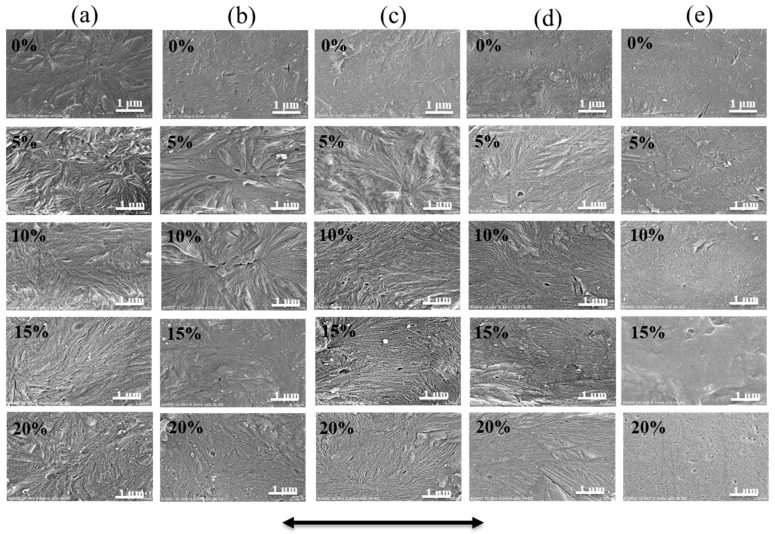
Scanning electron microscopy micrographs of the iPP sheet after stretching at different temperatures: (**a**) 110 °C, (**b**) 120 °C, (**c**) 130 °C, (**d**) 140 °C, and (**e**) 150 °C. The arrow indicates the stretching direction.

Scanning electron microscopy (SEM) micrographs illustrate a crystalline morphology is present during the solid-state stretching process. The micrographs of the iPP sheet are shown in Figure 8. When the initial iPP sheets were annealed at different temperatures, densely and randomly oriented β-modifications were observed. This shows that annealing could not promote the structural rearrangement of the molecular chains of the β-modification lamellae, and the crystal phase transformation process did not occur. When the stretching temperature was below the crystallization temperature (Figure 8a,b), a β-bundle crystal structure was observed [39,40,41], indicating that β-modifications dominated in the system. Oriented lamellae were not observed along the stretching direction, implying that the amorphous molecular chains were oriented, but that the lamellae were not stretched. This was because the external force was not sufficient to provide the energy to promote molecular chain movement. When the stretching temperature was 130 °C (Figure 8c), a cylindrite morphology could be observed when the stretching strain was between 10% and 20%. This can be explained by the fact that the molecular chains between the orientated lamellae and the partial β-modifications transformed into oriented cylindrites. Typically, a shish-kebab-like structure was produced due to slippage of lamellae in thermodynamic metastable β-modifications. Similar tendencies were observed in Figure 8d. Furthermore, it was found that the cylindrite structure at 130 °C was more stable, revealing that the regulation of macromechanical properties was greater.

The micrographs of the iPP sheet after solid-state stretching at 150 °C are shown in Figure 8e. The β-modifications almost completely disappeared, and the α-modifications dominated in the crystal region. This can be explained by the fact that most of the β-modifications melted, and the disordered molecular chains crystallized in the α-spherulite structure. This result corresponds to the XRD analysis results.

### 3.5. Mechanical Properties

**Figure 9:**
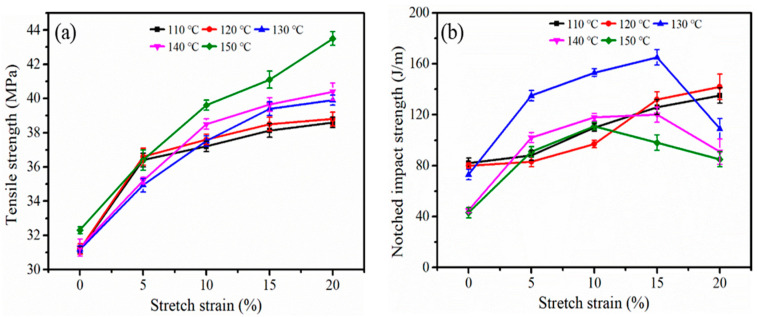
The tensile strength (**a**) and the notched impact strength (**b**) of the iPP sheet after stretching at different temperatures.

It is well known that the mechanical properties of iPP sheets are related not only to crystallinity, but also to the micromorphology of the crystal structure. The mechanical properties of the iPP sheet in this study at different stretching temperatures are shown in Figure 9. In Figure 9a, the tensile strength increased under increased stretching. When the stretching temperature was 150 °C, the tensile strength increased by 34% as the stretching strain increased to 20%. The molecular chain orientation promoted an increase in the total crystallinity, which resulted in an increase in tensile strength [42]. In addition, the β-modifications in the iPP sheet system were almost completely melted and recrystallized to α-modifications, which improved the tensile strength. Figure 9b shows the notched impact strength of the iPP sheet. It was found that the notched impact toughness increased with the increasing of the stretching strain when the stretching temperature was 110~120 °C. Thus, it was concluded that the molecular chains were oriented. When the stretching temperature exceeded the crystallization temperature, the notched impact strength of the iPP sheet after 15% stretching strain at 130 °C increased by 126% compared to the initial iPP sheet. According to the analysis results in Figure 5, there was a crystal phase transfer process in the system at this time, and a shish-kebab cylindrite structure existed. The metastable shish-kebab cylindrite structure could absorb more energy to transform into a stable crystal structure. Thus, the impact toughness was significantly improved [43]. When the stretching strain was 20%, the shish-kebab-like crystal phase transfer process did not dominate. As a result, the impact toughness was reduced. 

### 3.6. Cylindrite Structure Transformation Mechanism

**Figure 10:**
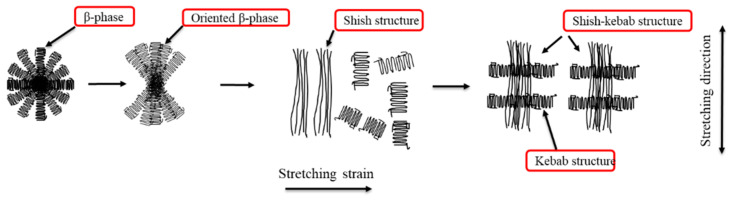
A schematic diagram reflecting the transformation of β-modifications for the stretched iPP sheet.

Upon deformation, the isotropic structure of polymer can be transformed into an anisotropic structure [28]. Hence, the transition mechanism and the relationship to the morphology and structure are important for polymer science. When the solid-state stretching temperature was higher than the crystallization temperature, the free energy of the crystallization of the molecular chain of the lamellar was low, and the folded lamellae were stretched and oriented. A schematic diagram reflecting the transformation of β-modification is shown in Figure 10. As the stretching strain was smaller, the application of an external force mainly promoted the disentanglement and extension of the amorphous-phase molecular chains, and the β-phase remained an isotropic spherulite structure. The inter-lamellar molecular chain underwent reversible elastic deformation, and no phase transformation occurred in this process. As the stretching strain increased, the β-phase lamellae was deflected and formed an oriented β-phase structure. As the stretching strain continued to increase, the β-phase lamellae was subjected to the persistent loading, extensive lamellae slippage inevitably caused the fragmentation of lamellae. In addition, part of the lamellae chains were pulled out. Subsequently, the lamellae chains were stretched and extended to form a shish-like structure [44,45]. RJ Young [46] suggested that when the spherulites were deforming homogeneously, the crystalline regions deformed by a combination of slipping, twinning, and martensitic transformation during the stage of deformation. As the stretching strain was higher, the fragmentation of lamellae started to rotate and surround the surface of the shish structure perpendicular to the stretching direction and formed a kebab structure, eventually forming a stable shish-kebab structure. Besides, random molecular chains were folded and grew epitaxially on the surface of the shish structure to form a shish-kebab structure.

## 4. Conclusions

This study investigated the deformation-induced crystal process of an iPP sheet containing the TMB-5 nucleating agent. The crystal orientation and the transformation of the iPP sheet were evaluated during a solid-state stretching process. β-modification lamellae were broken and a shish-kebab cylindrite structure was crystallized when the stretching temperature was above the crystallization temperature. Subsequently, the β–α transformation process occurred based on the existence of stress relaxation as the stretching temperature increased. The long period and the thickness of the crystal layer of the stretched iPP sheet were decreased during β-phase transformation into cylindrite. The crystal phase transformation process was conducive to improving the mechanical properties of the material. After 15% solid-state stretching at 130 °C, the notched impact strength of the stretched iPP sheet increased by 126%. Thus, we have significantly demonstrated that solid-state stretching of iPP sheets plays an important role, influences the morphology, and, as a result, can improve both the stiffness and the toughness of iPP.
